# Wearable Blood Pressure Sensing Based on Transmission Coefficient Scattering for Microstrip Patch Antennas

**DOI:** 10.3390/s22113996

**Published:** 2022-05-25

**Authors:** Mona K. El Abbasi, Mervat Madi, Herbert F. Jelinek, Karim Y. Kabalan

**Affiliations:** 1 Electrical and Computer Engineering Department, American University of Beirut, Beirut 1107 2020, Lebanon; kabalan@aub.edu.lb; 2 Electrical and Electronics Engineering School Department, Amity University Dubai, Dubai P.O. Box 345019, United Arab Emirates; mmadi@amityuniversity.ae; 3 Biomedical Engineering Department and Health Innovation Engineering Center, Biotechnology Center, Khalifa University of Science and Technology, Abu Dhabi P.O. Box 127788, United Arab Emirates; herbert.jelinek@ku.ac.ae

**Keywords:** miniaturized microstrip patch antenna, transmission coefficient scattering parameter, specific absorption rate, Moens-Korteweg equation, brachial artery radius-to-tissue thickness ratio, blood pressure

## Abstract

Painless, cuffless and continuous blood pressure monitoring sensors provide a more dynamic measure of blood pressure for critical diagnosis or continuous monitoring of hypertensive patients compared to current cuff-based options. To this end, a novel flexible, wearable and miniaturized microstrip patch antenna topology is proposed to measure dynamic blood pressure (BP). The methodology was implemented on a simulated five-layer human tissue arm model created and designed in High-Frequency Simulation Software “HFSS”. The electrical properties of the five-layer human tissue were set at the frequency range (2–3) GHz to comply with clinical/engineering standards. The fabricated patch incorporated on a 0.4 mm epoxy substrate achieved consistency between the simulated and measured reflection coefficient results at flat and bent conditions over the frequency range of 2.3–2.6 GHz. Simulations for a 10 g average specific absorption rate (SAR) based on IEEE-Standard for a human arm at different input powers were also carried out. The safest input power was 50 mW with an acceptable SAR value of 3.89 W/Kg < 4W/Kg. This study also explored a novel method to obtain the pulse transit time (PTT) as an option to measure BP. Pulse transmit time is based on obtaining the time difference between the transmission coefficient scattering waveforms measured between the two pairs of metallic sensors underlying the assumption that brachial arterial geometries are dynamic. Consequently, the proposed model is validated by comparing it to the standard nonlinear Moens and Korteweg model over different artery thickness-radius ratios, showing excellent correlation between 0.76 ± 0.03 and 0.81 ± 0.03 with the systolic and diastolic BP results. The absolute risk of arterial blood pressure increased with the increase in brachial artery thickness-radius ratio. The results of both methods successfully demonstrate how the radius estimates, PTT and pulse wave velocity (PWV), along with electromagnetic (EM) antenna transmission propagation characteristics, can be used to estimate continuous BP non-invasively.

## 1. Introduction

Hypertension is currently within the top ten causes of death worldwide. Blood pressure (BP) elevation is a common risk factor for arrhythmias, stroke and heart failure [1]. Although blood pressure is routinely measured during a healthcare visit, the traditional oscillometric method with a sphygmomanometer may cause discomfort to users and does not provide data about continuous variations in BP during either daily activities or at rest. Noninvasive intermittent techniques [2] are the most common methods currently in use, such as by ausculatory at-home devices. The at-home devices are simpler, quicker and require less expertise to use but are also often less accurate. They also provide, in most cases, only a one-time measure of BP, while arterial blood pressure fluctuates continuously. The available non-invasive, continuous BP monitoring devices include the arterial tonometry method, vascular unloading technique and pulse waveform characteristic parameter method which are all sensitive to movement, more expensive and need to be checked frequently for accuracy [3].

The proposed model measures S21 signals at two points between the first and second pair of antennas. The distance between the two antennas is 6.25 cm, which corresponds to half lambda. In this case, the antennas on the human arm work in the near field without any mutual coupling between them. The time shift delay via simulation between the distal (first pair of sensors) and proximal (second pair of sensors) transmission coefficient (S21) waveforms of both sensors is used to estimate PTT at different artery thickness-to-radius coefficients in analogy with the conventional methods in finding PTT from the Electrocardiogram (ECG) and photoplethysmography signals [4] at two different positions.

After PTT estimates were extracted as the time difference between proximal and distal S21 waveforms between the two pair of sensors, the Bramwell–Hill formula Equation (1) was applied to compute the PWV.

The regression routine of the mean BP reveals the correlation of the systolic and diastolic blood pressure conditions. The relationship between the PTT and BP (including systolic blood pressure (SBP) and diastolic blood pressure (DBP)) has been investigated and shows high correlation with the standard MK-BP estimation model.

## 2. Background

Recently, researchers have been focusing on EM wave sensing and microstrip patch antennas as a leading technology due to the EM interactions of scattering, reflection, absorption and transmission inside the human body that allows specific physiological features such as blood pressure to be determined. The microstrip patch antennas are metallic sensors that can radiate and receive EM waves. The properties of the reflected and transmitted EM waves, in terms of scattering parameters (S parameters), are used to determine and analyze the BP levels. S parameters ($S_{11}$, $S_{12}$, $S_{21}$ and $S_{22}$) are defined as the input-output relationship between terminals of the EM sensors [5]. The authors in [6] present a novel pressure sensor using a low-cost microstrip patch antenna placed at a distance from reflection plate foil. This paper indicates that when pressure is applied on the plate, the distance between patch and plate is reduced. It also outlined that the variation of the patch antenna’s resonant frequency is proportional to the change in pressure. However, this work did not interrogate the antenna into a real pressure circuit. Other researchers worked on a radiofrequency BP recorder as proposed by [5]. This approach relies on recording the blood flow velocity at the wrist using radio frequency Doppler radar technology to estimate the beat-to-beat BP. Accordingly, ref. [6] focused on designing two transmitting and two receiving antennas in parallel for the purpose of the non-invasive continuous blood pressure estimation method. The authors of [7] did not include the arterial blood pressure variations over time. The study only focused on the function of continuous radar by sending and receiving EM signals for pulse wave analysis for the purpose of pulse wave measurement.

A potential approach in estimating BP based on pulse transit time (PTT) used on body continuous wave microstrip patch antennas is proposed. Pulse transit time represents the time required for a pressure pulse to travel over an arterial length (L) [8].

The correlation between PWV and PTT can be illustrated by using the Bramwell–Hill formula and Moens–Koreteweg equations as:

(1) $$\mathrm{PWV}=\frac{L}{\mathrm{PTT}}=\sqrt{\frac{E\cdot h}{2r\rho }}$$

where *L* is the vessel length, *ρ* is the blood density, *r* is the inner radius of the vessel, *h* is the vessel wall thickness and *E* is the elastic modulus of vascular wall [8]. The elastic modulus *E* is given by the Hughes equation:

(2) $$E=E_{0}exp\left(\zeta P\right)$$

In [2], $E_{0}$ is the elastic modulus at zero blood pressure, *ζ* is a material coefficient of the artery and *P* refers to BP. Mukkamala et al. [9] reviewed the different models available to propose a relation model between PTT and BP. The main two problems associated with these models are that all PTT models require static calibration when using a cuff, in addition, most PTT-BP regression models depend on assumptions including flow, velocity and viscosity rather than on static standards. The model proposed in [10] addressed some of these issues associated with PTT models. Accordingly, they proposed an accurate small-sized bioimpedance measurement system for blood pressure monitoring that was tested on different blood vessel locations on the human body. The bioimpedance measurement system continuously reflects the change in BP through the change in the arterial cross-sectional area which is monitored by the change in arterial impedance. The proposed design provided good tracking of BP variations (PWV and PTT) with low power consumption in comparison to previous commercial pulse pressure sensors for the same level of accuracy. The main limitation of [10] is the large electrode size that may affect the current passing the artery, which may lead to a concentrated heating effect at the site of tissue contact.

This paper proposes a technology using a flexible and wearable microstrip patch antenna that analyzes the EM radiations out of a pair of metallic patch antennas of epoxy substrates to track patient BP continuously. This innovative BP measurement device is compact, lightweight, unobstructed and has low power consumption at about 8 mW for each metallic sensor.

## 3. Materials and Methods

### 3.1. Theoretical Equations

A major part of the EM electronic sensor is the antenna, which must be printed on flexible substrates for wearable applications that follow known design rules. The proposed antenna is designed based on a characteristic resonant frequency of 2.4 GHz ISM (Industrial, Scientific and Medical) band and an epoxy substrate of dielectric constant 4.3 for biomedical applications. The width of the proposed patch antenna can then be calculated as in [5]:

(3) $$W=\frac{C}{2\cdot f_{0}\sqrt{\frac{\varepsilon +1}{2}}}$$

where W is the width of the patch, C presents the speed of light, $f_{0}$ presents the resonant characteristic frequency and ε presents the value of substrate dielectric constant [5]. Both the air and the dielectric substrate have different permittivity; therefore, to consider this, the value of the effective dielectric constant ($\varepsilon_{eff}$) is calculated using (4). The value of $\left(\varepsilon_{eff}\right)$ is varied between 1 (dielectric constant of air) and the dielectric constant of the specified substrate (ε) [5].

(4) $$\varepsilon_{eff}=\frac{\varepsilon +1}{2}+\frac{\varepsilon -1}{2\sqrt{1\left(1+12\frac{h}{W}\right)}}$$

where h presents the thickness of substrate, and W is the width of the patch. The EM radiation excited by the patch passing through the ground also partially traverses the air and dielectric substrate. This fringing process maximizes the patch electrically.

(5) $$\Delta L=0.412h\left(\frac{\varepsilon_{eff}+0.3}{\varepsilon_{eff}-0.258}\right)\left(\frac{\frac{W}{h}+0.264}{\frac{W}{h}+0.8}\right)$$

Thus, the electrical length of the patch increases by 2Δ*L* [5].

(6) $$L_{actual}=L+2\Delta L$$

### 3.2. The Human Arm Model

**Figure 1:**
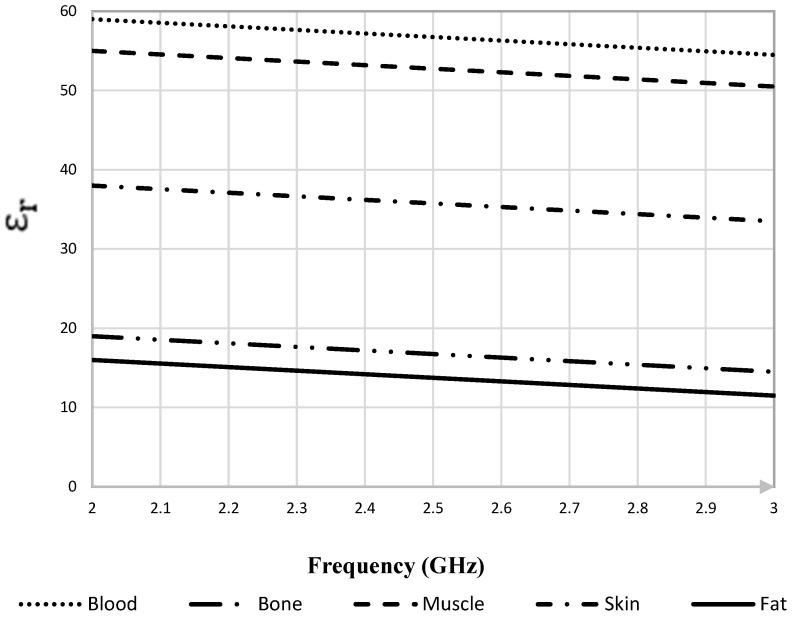
Relative permittivity ($\varepsilon_{r}$) in the frequency range from (2–3) GHz of the biological tissues selected for the heterogeneous simulated samples.

Since the human arm is a heterogeneous medium with layers of different tissues with different permittivities, it is necessary to study the dielectric properties of each tissue included in the model. The simulated human arm was chosen to be around a 45 mm radius for an average adult [11,12]. To show the variations in the relative permittivity ($\varepsilon_{r}$) of the various parts of the human arm: skin, fat, muscle, bone and blood over frequency range of 2 GHz to 3 GHz, Figure 1 is constructed. The variation of dielectric constant with frequency range allows reaching the targeted veins and arteries through the skin, muscle, bone, blood and fat tissue layers while maintaining good sensitivity. This variation impacts the acquired signal, it also affects the sensing wall thickness to vessel radius ratio (h/R).

Table 1 clearly shows that the blood has the highest permittivity, whereas fat and bone have similar permittivities. It also presents the different electrical permittivity ($\varepsilon_{r})$ and conductivity σ (S/m) properties in addition to the different mechanical compressibility $\sigma_{mec}\;\left(W/K/m\right)$ and the mass density $\rho \;\left(\mathrm{Kg}/m^{3}\right)$ properties of each tissue over the frequency range of 2–3 GHz [12,13,14].

**Figure 2:**
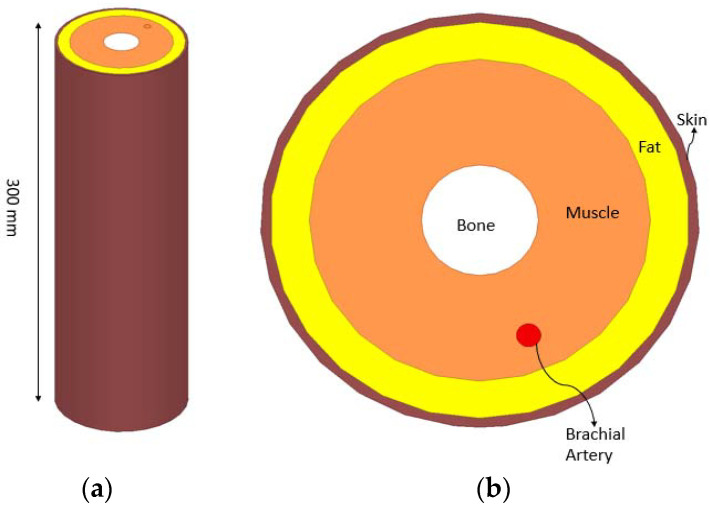
Cylindrical modeled multi-layer human arm model developed in ANSYS HFSS: (**a**) perspective view; (**b**) cross section of the top view (zoomed in).

The simulated arm model is designed in HFSS, as shown in Figure 2, and consists of skin, fat, muscle, bone and arterial tissues.

The artery is reasonably modeled to mimic the properties of the brachial artery, for which simulation has been performed using five different diameters 4.5 mm, 4.6 mm, 4.7 mm, 4.8 mm and 4.9 mm, which has also been reported by previous work [13,14].

This implemented model is reliable because it is close to the voxel model that is capable of capturing a high-resolution level of anatomical detail and is found to be cheaper and less complex when carrying out electromagnetic simulation tests for wearable antennas. It also specifies the dielectric properties of the different human tissues based on the operating frequency.

### 3.3. Design of the Proposed Microstrip Patch Antenna

A compact rectangular inset-fed antenna with a 50 Ω microstrip feed line and a waveguide (partial ground plane, substrate and radiating patch) was designed and optimized in an ANSYS HFSS environment.

**Figure 3:**
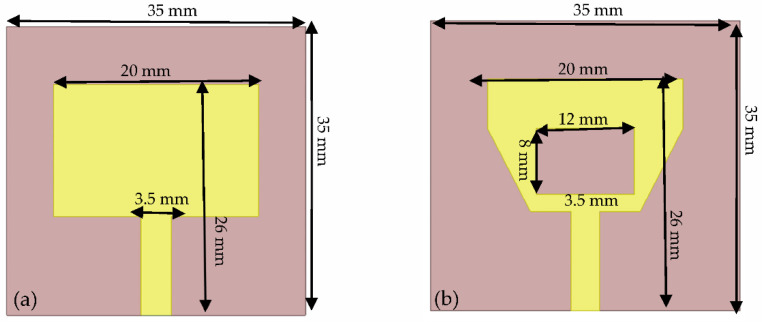
(**a**) Conventional microstrip patch antenna versus (**b**) the proposed patch antenna.

The antenna is inspired from the literature [15]. Although the microstrip antenna implemented in [15] is flexible and dedicated for medical applications, it achieved resonant frequency in free space and with no curvature is 2.3 GHz. The proposed antenna is proved through simulation and measurement to attain a resonant frequency of 2.4 GHz. This is achieved as shown in Figure 3, by truncating both sides of the antenna, creating a hole at the center and changing the substrate material resulting in resonance at the targeted frequency of 2.4 GHz. The proposed antenna geometry as shown in Figure 3, achieves a narrow band with good matching properties at 2.4 GHz. The narrow band radiator antenna focusses its power on the signal of interest while tuning out any interferences. Moreover, the receiving antenna can cancel out unwanted wideband noise [15].

Indeed, the full ground plane in the initial design [15] is replaced by a partial ground plane, in order to shift resonance from 2.2 GHz to the targeted ISM frequency of 2.4 GHz and maintains a narrow band. Note that the antenna has an omnidirectional radiation pattern. Thus, the designed antenna is small in size, robust, low cost, consumes a small amount of power, comfortable to wear and is easy of manufacturing. Such antenna geometry is suitable for body-worn applications.

Antenna dimensions were optimized, as shown in Table 2, according to the theoretical design rules mentioned before.

**Figure 4:**
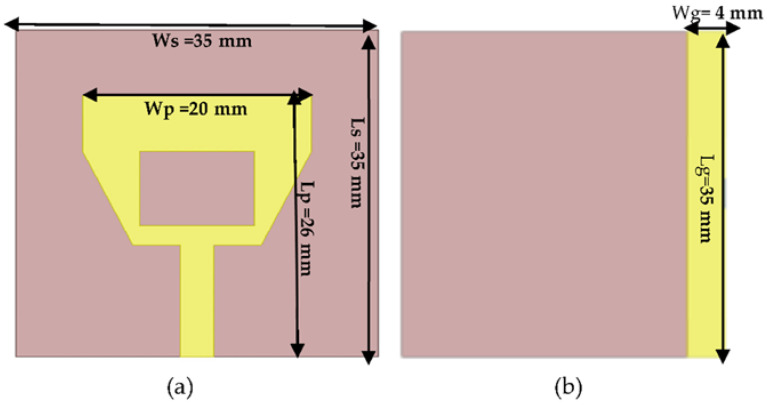
Designed flat patch antenna on epoxy flexible material: (**a**) front view and (**b**) bottom view.

The epoxy substrate was of 0.4 mm thickness (h), of 35 × 35 mm area, and the dielectric constant and dielectric loss tangent were ε = 4.3 and tanδ = 0.004, respectively. Epoxy substrate was utilized as it can be used across curved surfaces. In addition, it is low-cost, lightweight and has high surface energy [15]. The schematic of the proposed antenna is shown in Figure 4.

**Figure 5:**
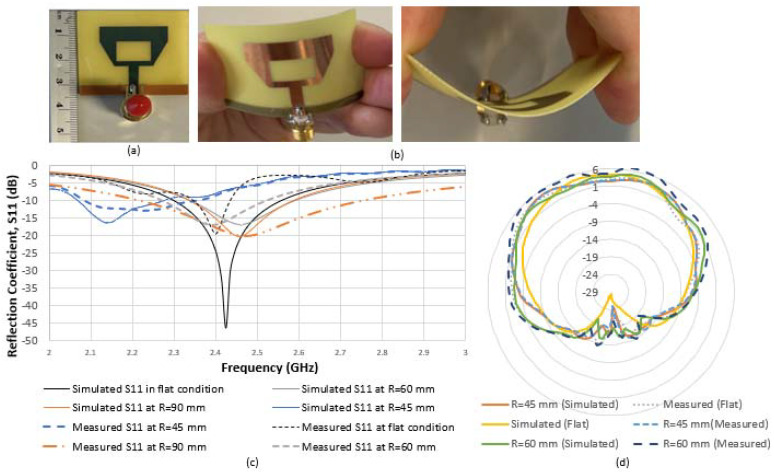
Structure and performance measurement of the fabricated patch antenna: (**a**) schematic of fabricated antenna with coaxial feed on a flexible substrate; (**b**) photos of different antenna bending angles at 20° and at 60°; (**c**) variation of measured (dotted lines) and simulated (solid lines) reflection coefficient for different bending angles at 20°, 40° and at 60° mm; (**d**) simulated and measured off-body 2-D radiation patterns at 2.4 GHz of the proposed antenna in flat and at different bending conditions.

The antenna operating at 2.4 GHz, exhibits high gain, high directivity, good electrical efficiency and noticeable detection range. Figure 5a,b compares the fabricated planar antenna using a coaxial feed to the bent antenna over a cylindrical shape with different radii, which also accommodates different human curvatures. As shown in Figure 5c, the antenna is measured in free space as a reference, resulting in a reflection coefficient ($S_{11}$) of −45 dB at 2.42 GHz with a high radiation efficiency.

Implementing a curved antenna led to a minimal shift of the resonant frequency and reduced return loss for all antenna curving conditions at 20°, 40° and at 60°. The antenna maintained a stable performance for several configurations over planar or curved surfaces with minimal difference between the flat and bent *S*_11_ response of the antennas. The corresponding radii resemble the different human arm circumference options, which the antennas are placed on for measuring BP. We can conclude that the antenna operates normally with an acceptable operational bandwidth. Figure 5d shows a good monopole 2D radiation pattern for planar and bent antennas. As expected, the patterns show that the angular width (3 dB) tends to decrease along with the increase in bending of the antenna. As a result, the antenna is well suited for wearable applications.

Table 3 shows the increment of return loss (*S*_11_) as the bending increases; that is as a function of the radius R. Generally, a larger return loss results in more energy delivered into the antenna. Therefore, the antenna must be designed with a wide frequency range. The wider the bandwidth, the better the antenna performance.

**Figure 6:**
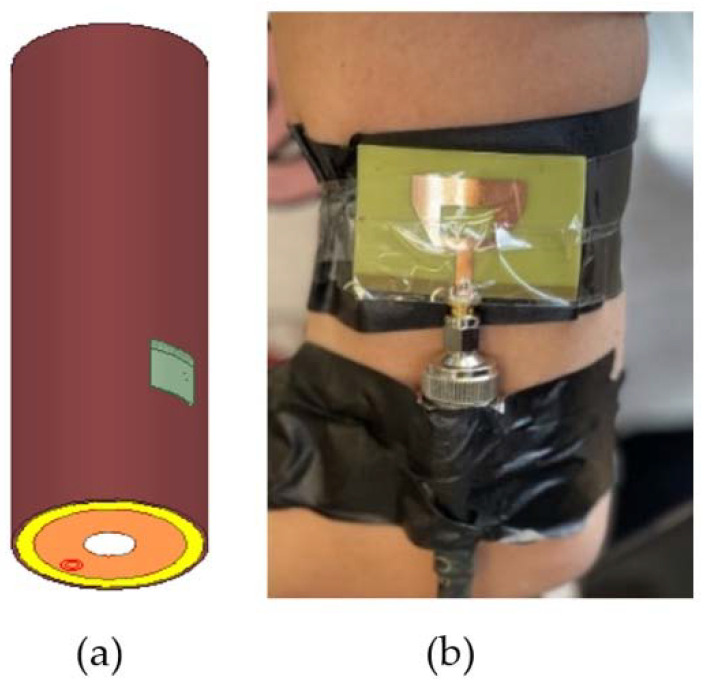
The microstrip patch antenna on the simulated human arm model of (**a**) 45 mm radius versus (**b**) the fabricated microstrip patch antenna on real human arm subject of the same radius.

Body tissues are extremely lossy and have a high dielectric constant and electrical conductivity, which results in a large loss in the RF signal strength transmission from the body to free space. Therefore, as the human body absorbs a large amount of radiated electromagnetic power, it is normal for the antenna characterizations to significantly decrease. Thus, the resonance frequency will shift to a higher band. When the antenna is bent around the simulated human arm model of radius 45 mm, the resonant frequency is shifted to 2.6 GHz with *S*_11_ at about −20 dB. This is the same case as shown in Figure 6, when the fabricated patch was placed on a real human arm subject with the same radius of 45 mm. As shown in Figure 6, the measured reflection coefficient is decreased due to effects of impedance mismatching of the lossy human tissue that provides the dielectric loading.

However, the measured results show some degradation in the performance of the antenna. Thus, for better results, future research should add a layer with relative permittivity equal to the average permittivity of the arm tissue (skin, muscle and bone) and thickened equal to the average diameter of the human arm. In this case, the general results and accuracy will be improved.

**Figure 7:**
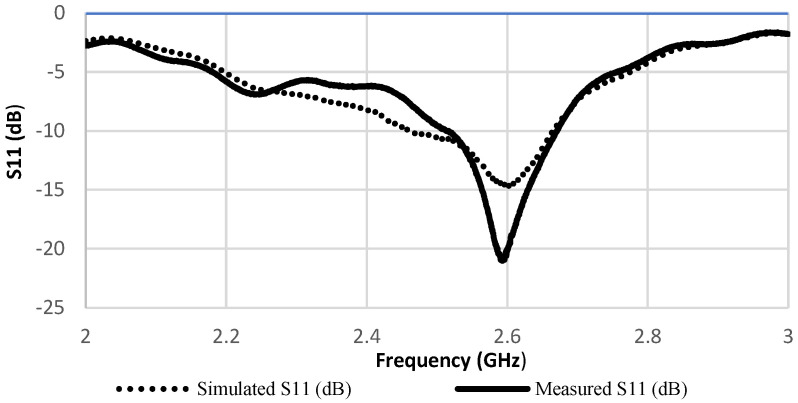
Simulated return loss with the presence of the simulated human arm model versus measured return loss on a real human arm of the same radius 45 mm as simulated.

Thus, considering the dielectric properties of human tissues, there is an acceptable penetration ratio of the antenna into the body, which is verified by the good impedance matching obtained from the simulation and measurement procedures as shown in Figure 7.

According to [15], antenna bending will induce a shift in resonant frequency. Moreover, additional shift will occur in the presence of a human body [16]. Furthermore, though the resonance was obtained at 2.6 GHz (Figure 7) near the arm with maximum bending and the design was optimized at narrow band 2.4 GHz in free space without bending, the study relies on obtaining the scattering parameters S21 of the two antenna pairs over [2,3] GHz. The dielectric properties of the human arm phantom were updated each time the frequency of the antenna is varied over [2,3] GHz because they are frequency dependent [17] and are presented Figure 1.

However, the measured results show some degradation in the performance of the antenna. Thus, for better results and as a future work, adding a layer with relative permittivity equal to the average permittivity of the arm tissue (skin, muscle and bone) and thickened equal to the average diameter of the human arm. In this case, the general results and accuracy will be improved.

The human body is of high permittivity, known as a dispersive material, leading to a large amount of EM radiations being absorbed out of wearable antennas. Therefore, it is, necessary to evaluate the Specific Absorption Rate (SAR), which is equal to the square of the electrical field multiplied by conductivity and divided by mass density [17]. It is a measure of how much power is absorbed per unit mass of a conductive material, and is defined as:

(7) $$\mathrm{SAR}=\frac{\sigma {\left|E\right|}^{2}}{\rho }$$

where σ is the electrical conductivity of the material, *E* is the RMS magnitude of the electric field at a given point and ρ is the mass density of the material [17]. Specific absorption rate results must conform to the measurement standards specified by the council of IEEE safety standards of 4 W/kg averaged over 10 g of actual tissue [18,19]. Otherwise, adverse biological effects can result due to a large amount of electromagnetic power absorbed [18].

**Figure 8:**
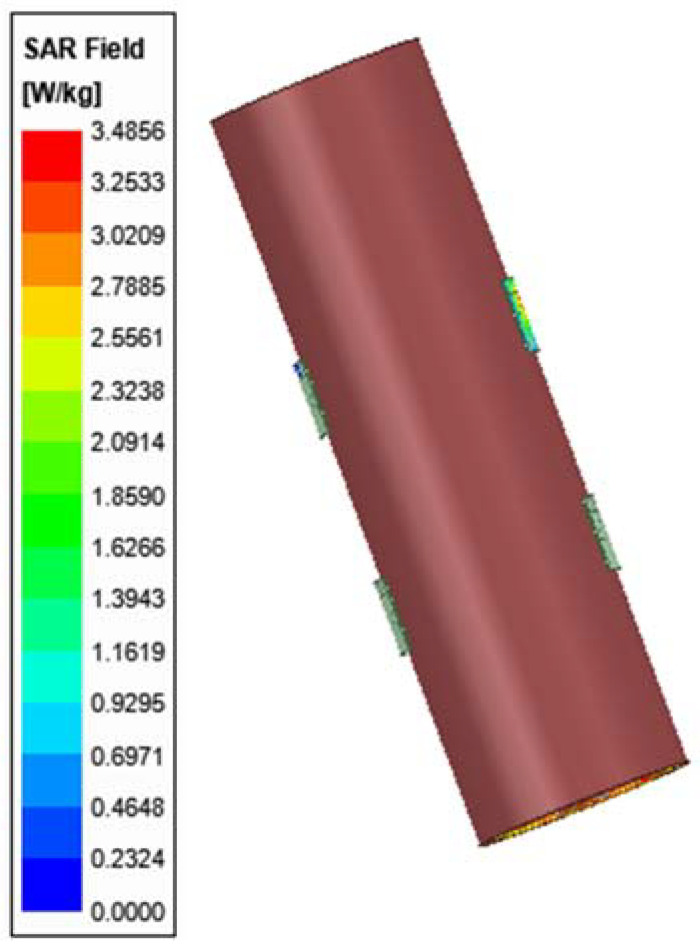
Simulated SAR of on-body arm model.

In this regard, Figure 8 shows how the HFSS software can be a particularly useful tool for calculating SAR for tissues. It is found that the safest total transmitted power of the pair of transmitters is 3.48 W/Kg, which complies with IEEE-standard for an average human arm of 10 g.

To study the behavior of radio waves in HFSS, the simulated human arm model that was created previously can be a viable candidate for SAR evaluation as shown in Figure 8. For the characteristic resonant frequency, the range of simulated source power was 1 W to 50 mW. To examine how SAR levels of the arm tissues change depending on the source power, the simulations performed at 2.4 GHz were repeated simultaneously.

The SAR values for 10 g arm tissues, obtained using the HFSS, are listed in Table 4. During the simulations, it was observed that the blood and the muscle absorbed more energy than any other tissue. Since the density and conductivity of bone is low, it has the least energy absorption of the tissues. Thus, the decreased input power required results in low SAR levels which is critical to this study, since the antennas have continuous radiation wave exposure that penetrates deep into the human arm [19].

### 3.4. The Proposed Blood Pressure Determination Method

**Figure 9:**
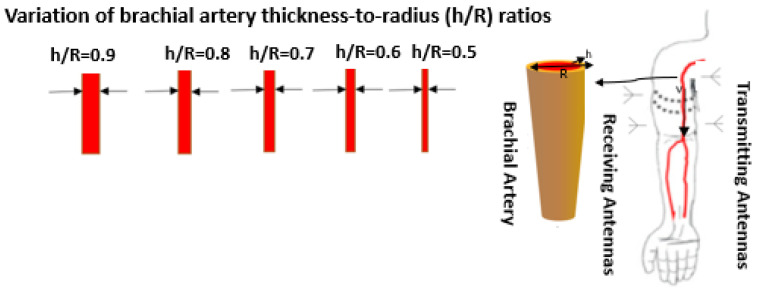
Illustration of proposed method related to brachial artery h/R ratio variation in respect to blood pressure estimation. Healthy elastic arteries = Normal BP and elastic artery stiffening = increase CV risk. The arterial vessel is often modeled as an elastic cylindrical tube. The simulation would be performed at five instances of brachial artery diameters to mimic the condition of blood flow. Although the above simulation model lacks blood flow, the consideration of different arterial diameters partly compensates for the hemodynamic effects. The choice of different arterial diameters complies with the characteristics of the arterial system. It was reasonably assumed that the brachial artery diameter changes uniformly at each dielectric characteristic change of the flow of blood, fat, muscle, bone and skin.

There is no exact and direct relation between EM wave propagation and pressure. We relied on the development of a wearable and conformal antenna that can sense and monitor pressure or any environmental factors. Meanwhile, the developed relationship between transmitting and receiving antennas depends on the change of reflected EM waves out of the antennas and relates to the change in artery radius and thickness. This means any change in the properties of (h/R) ratio of the artery can be determined by the change in the shape of electromagnetic radiation waves between the pair of antennas and can be indirectly related to variations in blood pressure levels [20,21]. Considering that a larger brachial artery radius is associated with a greater risk of elevated levels of blood pressure [22,23], the new proposed topology is based on two critical factors to detect the variations in blood pressure: (i) the change in the brachial artery (BA) thickness-to-radius specifications as described in Figure 9; and (ii) the change in dielectric constant.

Blood is commonly used as a good conductor of electricity [24], therefore modeling the electromagnetic changes associated with blood flow plays a significant role in assessing hypertension. Blood flow modeling was performed by considering the main artery of the upper arm and the brachial artery (BA) modeled as an elastic tube. Because there is no blood flow option to model during simulations, we have taken different values of h/R to mimic the blood flow characteristics.

The assessment of the arterial wall (h/R) ratio or brachial hardening has a prognostic role in the prediction of hypertension and the brachial artery is a good reflector of central blood pressure [24,25]. The brachial artery is a good reflector of central blood pressure [22,23] as its diameter varies periodically with the heartbeat. The low power transceiver design (TX/RX antennas) radiates electromagnetic waves. The EM waves emitted from the EM microstrip patch antennas are transmitted through the human arm model and are affected by the different conductivity and permittivity tissue layers ranging from 2 GHz to 3 GHz. The latter goes deeper into the skin, fat, muscle, bones, veins and arteries. In the same manner, these radiations are tested carefully and are proven to be safe and effective on the human body without any harmful effects.

In short, the variations are transmitted and reflected in the form of waves, leading to an effective relationship between the transmission coefficient, pulse wave velocity and the change in arterial diameter. The general idea illustrated in Figure 9, is that while the brachial artery diameter reacts to changes in central blood pressure, this diameter can be sensed using the antenna system. Large arterial diameters imply increased blood flow and hence, the result is a higher blood pressure. The dielectric constant and conductivity of the tissues varies substantially with frequency and can therefore only be considered constant over a finite range of frequencies.

## 4. Results

### 4.1. Transmission Coefficient Scattering Parameters of Antennas

**Figure 10:**
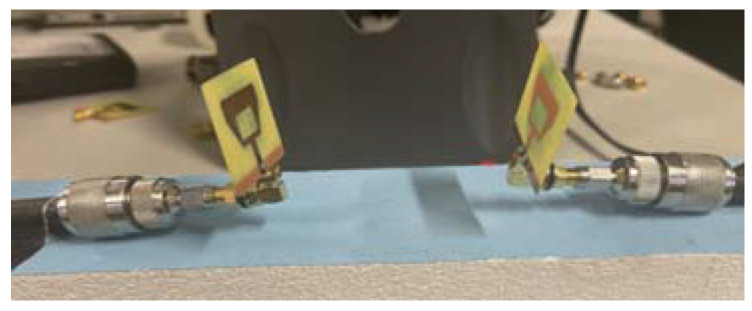
Two fabricated antennas in free space separated by 6.5 cm.

To determine the transmission coefficient scattering parameters of antennas, two microstrip patches were fabricated and placed 6.25 cm apart in free space as shown in Figure 10.

**Figure 11:**
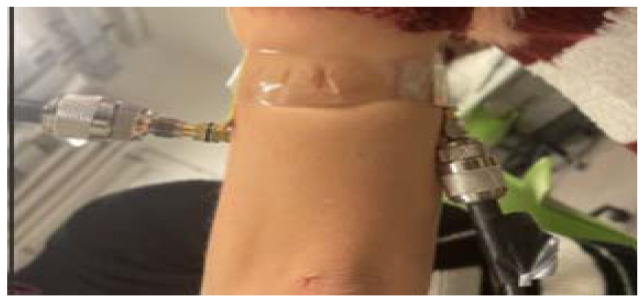
The fabricated pair of antennas are placed on a real human arm of radius 45 mm to examine the transmission coefficient.

The distance must be at least a half wavelength to avoid mutual coupling [20]. In this setting, the patch functions by emitting continuous RF waves and the receiving patch captures these waves. The properties of the reflected waves and the transmitted waves, in terms of scattering parameters or S parameters, were used and analyzed to determine the variation in blood pressure. To analyze the possible performance of antennas on a human arm, the transmitting antenna is attached to one side of the upper human arm and the receiving antenna is attached to the opposite side as shown in Figure 11.

### 4.2. Electric Field Distribution

**Figure 12:**
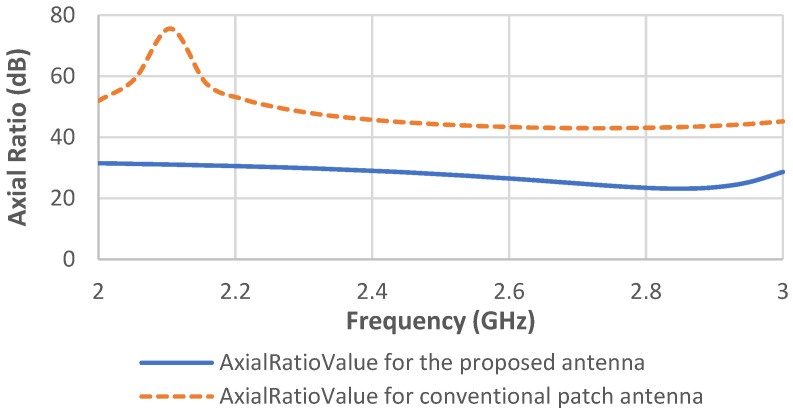
Axial ratios of the conventional and proposed patch antennas.

The axial ratio is the ratio of orthogonal components of an E-field. Figure 12 shows that the axial ratios of the two antennas before and after modification is above 3 dB. Therefore, both antennas have linear polarization.

**Figure 13:**
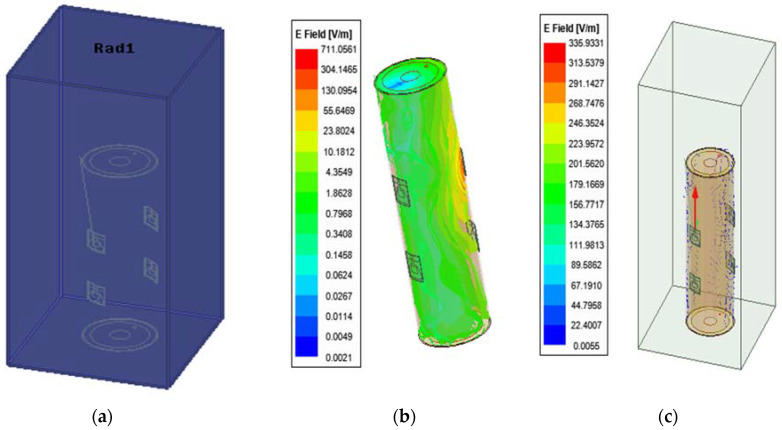
Phantom boundary conditions: (**a**) absorbing boundaries; (**b**) Simulated total electric field strength at the absorbing conditions; (**c**) scattering electric field strength at the absorbing conditions.

Figure 13 shows the electric field distributions of the proposed patch antenna at 2.4 GHz. The direction of propagation of the radiated waves is perpendicular to the human arm surface, whereas the electric field is linearly polarized, and its direction is parallel to the human arm. It is shown from Figure 13 that half of the electric field is lost as scattering waves. However, there is an acceptable value within the modeled brachial artery.

### 4.3. Measuring Transmission Coefficient Scattering Parameter and Computing Blood Pressure

**Figure 14:**
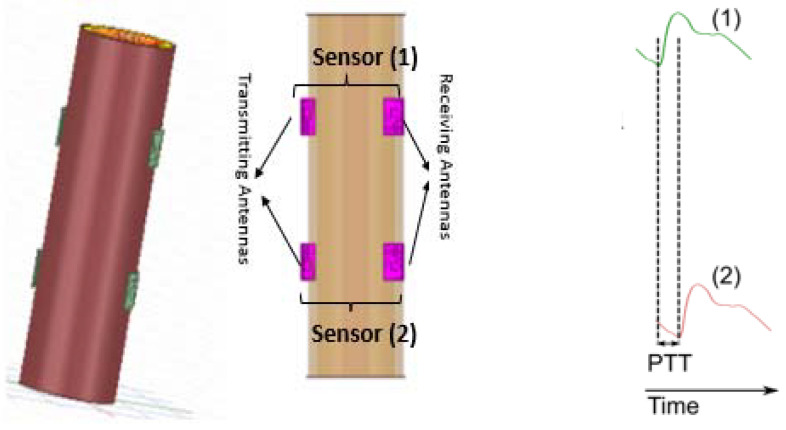
Two pairs of transmitting and receiving antennas bent over a cylindrical multi-layer human arm model developed in ANSYS HFSS. Electromagnetic waves leaving the antennas can reach the targeted brachial artery along with the wide dielectric characterization range. L = length from the first sensor to the second; PTT = time taken for the pulse wave to propagate from: (1) the upper side; to (2) lower side of the brachial artery. Based on the methodology of this study, the PWV-PPT relationship was noted for three distinct cases reflecting the effect of blood flow, and thus, blood pressure estimation. The cases were investigated with different artery h/R ratios of 0.5, 0.7 and 0.9.

Pulse Transit Time (PTT) is known as the time delay between two locations of an artery [26]. It has received much attention over the recent decades because of its capability to evaluate Pulse Wave Velocity (PWV), and hence, track BP change [27]. Utilizing PTT could permit non-invasive, continuous automatic and cuffless BP monitoring. The current approach uses two pairs of sensors as shown in Figure 14, placed at the brachial artery of the human arm model (upper arm and lower arm).

**Figure 15:**
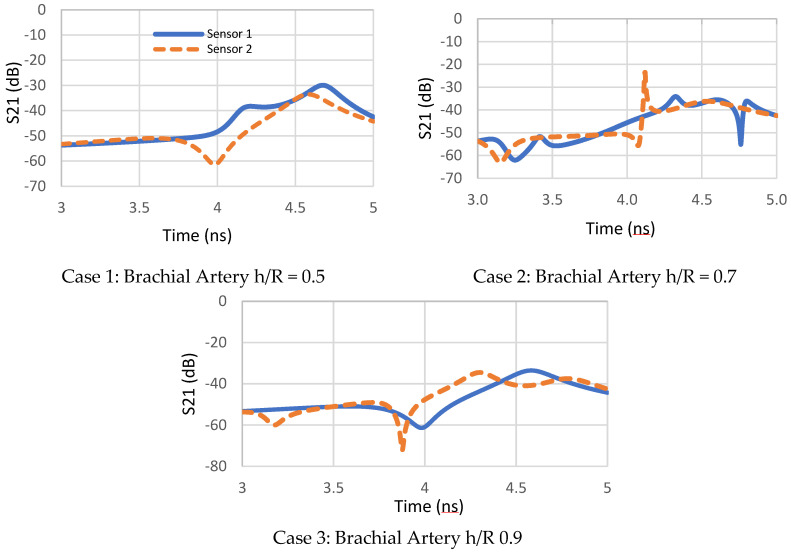
Three cases of simulation results of transmission coefficient variation in the time domain for the human artery of h/R ratios = 0.5, 0.7 and 0.9.

Placing both sensors at the central part of the brachial artery, a communal problem of measuring peripheral BP values is avoided. The changes in artery blood volume characteristics are modeled as changes in blood artery diameters. Two separate pulse recordings over a known distance were obtained. Hence, the distance (L) between the two sensors allows calculation of PWV as indicated in (Equation (1)). The innovative method is illustrated, by the following factors:

1. The time shift delay shown in Figure 14 between the two waveforms of both sensors is used to estimate PTT at different artery thickness to radius coefficients.
2. The transmission coefficient waveforms between the two pairs of antennas are noted through three main simulations of brachial artery h/R variation for the same human arm model, as shown in Figure 15.

The coefficient S21 with such lossy material, at 2.4 GHz, is extremally small, but it is large enough to be larger than the noise, and therefore, the changes in the S21 parameter are credible.

**Figure 16:**
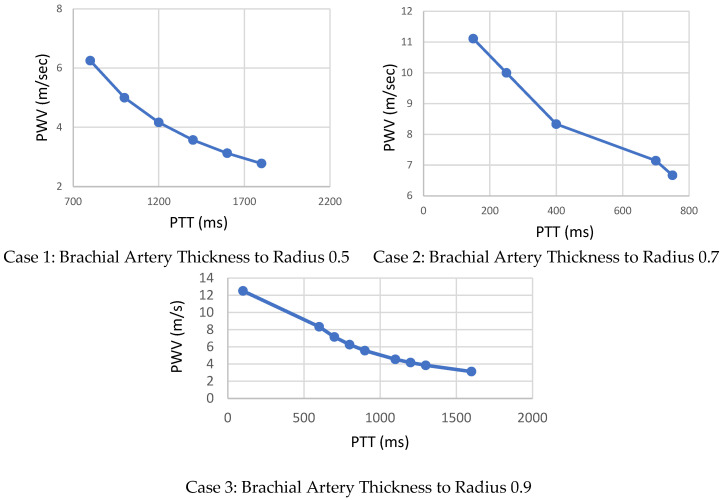
Simulation results of PTT-based pulse measurement dual sensors: Through this variation, PTT estimates for human artery of thickness/radius ratio h/R = 0.5, 0.7 and 0.9. Pulse transit times are extracted and then PWV can be calculated to provide a comprehensive overview of the blood pressure variation level.

**Figure 17:**
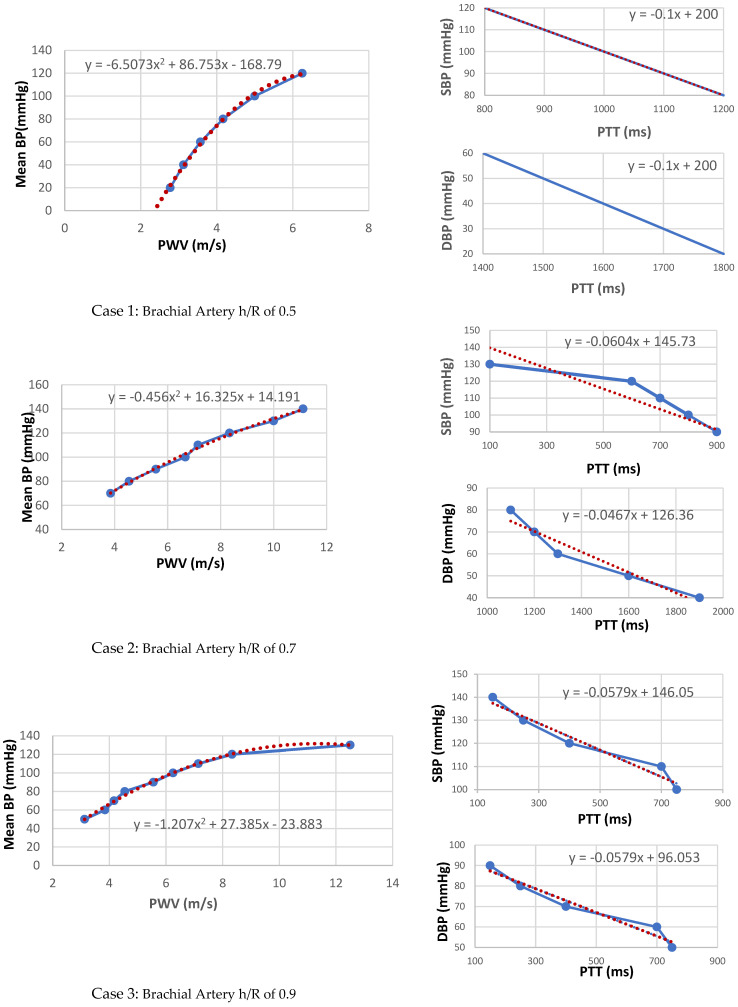
Mean BP versus PWV for the brachial artery of h/R ratios of 0.5, 0.7 and 0.9, with linear regression trend line. In addition, the relationship of PTT to Systolic BP with a straight line representing linear regression and the relationship of PTT to Diastolic BP with a straight line representing linear regression.

3. The distance (L) between the two sensors allows calculation of PWV as shown in Figure 16. After PTT estimates were extracted as the time difference between proximal and distal transmission waveforms between the two pair of sensors presented previously in Figure 8, the Bramwell–Hill formula was applied to compute the PWV from the distance and pulse transit time (PTT) as shown in Figure 11. Pulse transit time is, in turn, estimated by acquiring proximal and distal arterial waveforms from the two sites and then detecting the foot-to-foot time delay between the waveforms [26,27,28,29]. The PWV-PTT relationship is noted in the three different brachial artery h/R ratio changes. The arterial thickness-to-radius ratio varies with the blood vessel volume variation, and thus, arterial BP. The accuracy of BP estimation is affected if the arterial diameter is ignored. Pulse wave velocity can be calculated based on the Bramwell–Hill formula and is therefore indirectly related to arterial distensibility as shown in Figure 16.

4. Arterial distensibility is related to mean arterial pressure through regression analysis that reflect trends in blood pressure over the longer term as well as indicating abrupt changes in arterial systolic and diastolic blood pressures as shown in Figure 17.

Whilst the PWV and PTT levels were set at normal values. The regression routine is shown in Figure 17 as a red dotted trend line of the mean BP, and it reveals the correlation of the systolic and diastolic blood pressure conditions. However, as the varying brachial artery coefficients increase, there is an increase in blood pressure levels [30,31,32]. For the change in transmission coefficient curves of brachial artery h/R ratio of 0.7, the systolic blood pressure decreased from 120 mmHg to 90 mmHg.

To our knowledge, the relationship between electromagnetic waves, PTT and arterial pressure has not been previously systematically studied. This is an innovative step that demonstrates the relationship between the PTT, PWV and BP in detail through simulations, based on varying the arterial specifications. This method requires no calibration. As the arterial pressure changes increased, the PTT decreased significantly. The maximal PWV of 11 m/s corresponds to high systolic and diastolic blood pressure levels. Non-linear curve fitting (Figure 17) using MATLAB to estimate the systolic and diastolic blood pressures of the collected data. Table 5 shows a comparison summary of the estimated PTT, PWV and arterial BP of different arterial h/R ratios. The highest brachial artery h/R ratio presents the highest PWV and consequently the highest levels of blood pressure.

### 4.4. Comparing the Proposed Method to the Standard MK Model

The Moens–Korteweg (MK) given in Equation (1), is used to prove the previously proposed innovative model. The MK equation applied many of the assumptions that do not hold for human arteries. The general aspect of this analytical method is reliable for continuous, cuffless and noninvasive blood pressure monitoring techniques. The pressure pulse propagation speed passing along the artery is a function of its elasticity and pressure with the underlying condition that brachial artery geometries are dynamic. The condition is based on the variation of the h/R ratio for a healthy person (0.08 to ~0.22) before artery deformation [22,23,25] and increases significantly after deformation to reach 0.5 to ~0.9 for a person with an arterial disease [22,23,25]. The artery deformation is due to the variations in the blood pressure levels. Another important application of the MK equation is to determine the arterial strain “E_inc_” because of its application in the prediction of elevated blood pressure levels. The E_inc_ is also an intrinsic measure of arterial wall stiffness [31,32]. The connection is shown to be due to the elasticity of the artery walls because any increase in pressure will lead to an increase in the artery cross-section [32]. Thus, there is a positive correlation between the brachial artery elastic properties, the thickness and the artery radius, the arterial strain (E) and the PWV as specified in Equation (1).

The steps of this method are summarized as follows:

1. The MK model is established to compute the PWV. The PWV depends on the elastic properties of both arteries and blood;
2. The arterial strain (E) can be computed from the Hughes Equation (Equation (2)) where the considered blood pressure range lies between 5 kPa to ~20 kPa;
3. The h/R of the arteries are dynamic and can change up to 30% with changes in blood pressure;
4. Table 6 shows that PWV varies directly with the arterial wall stiffness and is related to the wall thickness and elasticity and inversely related to arterial radius. It has been shown that any change in arterial radius is related to changes in instantaneous blood pressure.

Table 7 presents a summary of PWV and artery stiffness (E) estimates for different values of h/R ratios after deformation due to blood pressure changes. It also indicates that at certain arterial ratios, high PWV values, and thus, high blood pressure persists. Large arteries that present high pulse wave velocity, however, gradually lose their elasticity and become stiffer. This leads to an increase in blood pressure levels.

The second method relies on the MK equation to prove and justify the overall approach. By observing the increase in PWV in terms of an increase in arterial wall thickness, a simple analytical relationship can be computed.

**Figure 18:**
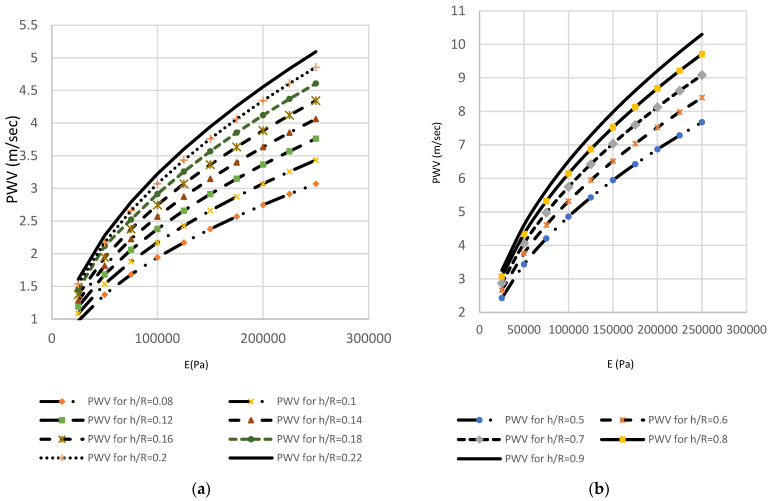
Effects of wall thickness divided by vessel radius in the brachial artery (before and after deformation) on PWV. (**a**)The theoretical, i.e., Moens–Korteweg, relationship between the PWV versus artery stiffness (E) for a human brachial artery before deformation; (**b**) the theoretical, i.e., Moens–Korteweg, relationship between the PWV versus artery stiffness (E) for a human brachial artery after deformation due to blood pressure. It is deduced that PWV increases with increased arterial stiffness.

**Figure 19:**
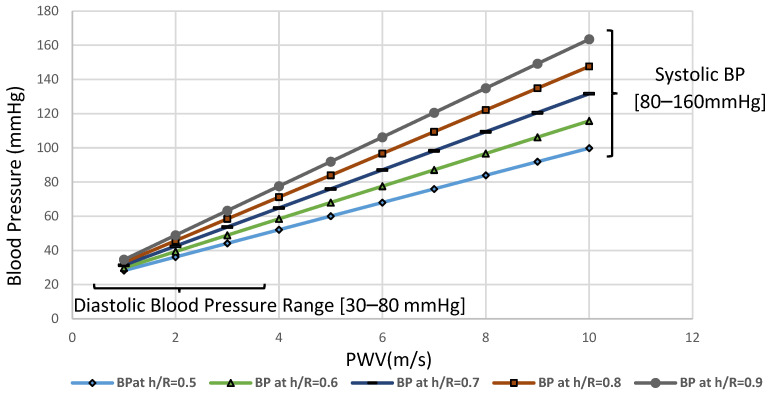
Blood pressure P versus normalized PWV for human brachial artery. The absolute risk of arterial blood pressure increased in relation to artery PWV.

**Figure 20:**
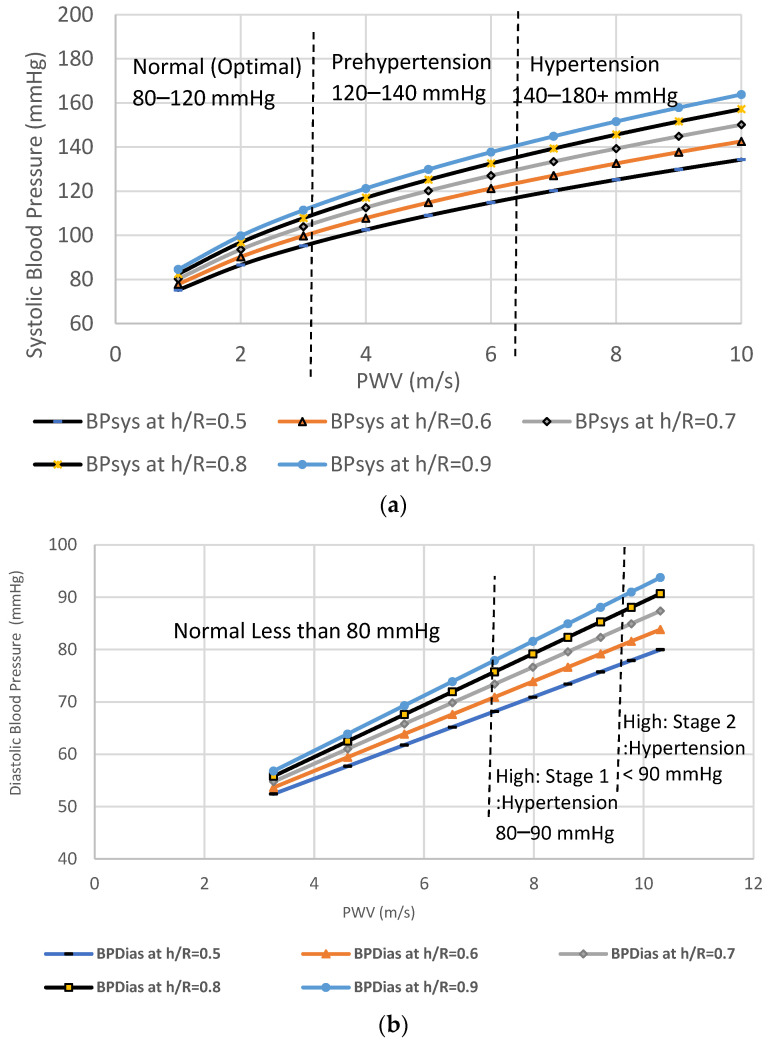
(**a**) Relationship between pulse-wave velocity and systolic blood pressure (in mmHg); (**b**) elationship between pulse-wave velocity and diastolic blood pressure (in mmHg).

Using the mathematical “least square approach” analysis to determine the relation between BP and PWV, BP can be represented by the quadratic equation:

(8) $$\mathrm{BP}=a*{\mathrm{PWV}}^{2}+b$$

where a and b are scaling coefficients that depend on the material properties and geometry of the artery. The quadratic Equation (6) can be divided into a system of two equations: the DBP and SBP equations. Therefore, ${\mathrm{BP}}_{\mathrm{Dia}}$ and ${\mathrm{BP}}_{\mathrm{Sys}}$ can be estimated according to the blood flow information by deriving linear equations where:

$a_{1}$ and $a_{2}$ are weighting factors of PWV, and the linear basis variables $b_{1}$ and $b_{2}$ are statistically calculated from population data with diverse levels of BP. Thus, the weighting factor $a_{1}=0.84$ and $a_{2}=0.66$ and the basis variables $b_{1}=6.39$ and $b_{2}=5.29$, were applied in this study. Model coefficients can be determined using a least-squares fit from the graphs and the data pairs that are found to be $a=0.18\;\mathrm{kPa}\cdot s^{2}\cdot m^{-2}\;\mathrm{and}\;b=2.7\;\mathrm{kPa}$. Figure 18 defines a linear relationship between the increase in PWV and blood pressure (kPa). As shown in Figure 18, these findings suggest a cause–effect relationship between h/R ratio of the brachial artery, increase in the pulse wave velocity, and hence increase in the levels of blood pressure. As the level of blood pressure increases, as shown in Figure 19, an increase in PWV follows. Then, the pressure measured in kilopascals can be converted to a mmHg reading by letting 133.322 kPa equal one mmHg [32]. At high vessel diameters, the high wall thickness corresponds to higher levels of blood pressure. Figure 20 depicts the dependence of PWV on blood pressure for the Systole Phase (marked as “SBP”) and a Diastole Phase (marked as “DBP”) for the vessel of different thicknesses of a human artery. It is well noted from Figure 20 that the nearly linear increase in the h/R ratio results in increased systolic and diastolic pressure. Thus, this second mathematical approach indicates that any change in arterial cross-sectional area is directly proportional to a change in BP. Predicted effects of driving pressure on h/R ratios are consistent with the simulated two pairs of antennas. Briefly, we found that arterial stiffness, as indexed by PWV, is a dependent predictor of the longitudinal increase in SBP and DBP.

Table 8 shows the summary of the two methods. There is a good consistency between the results of the two methods. According to the results, by comparing the PWV, SBP and DBP values, both models show good agreement and high accuracy in a systematic and detailed algorithm.

## 5. Discussion & Conclusions

There is no previously published information regarding the estimation of BP from the output of two microstrip patch antennas placed on a human arm. In this paper, we outline the use of a microstrip patch antenna on a simulated human arm and also tested the antenna on a human arm subject. The result shows that the performance of the proposed antenna is dependent on the extent of antenna bending but still operates efficiently within the expected curvature of the human arm for measures of brachial artery BP. The findings further highlight the importance of taking into account accurate dielectric properties of the constituent tissues in the estimation of EM medical technologies. The specific absorption rate was also analyzed and confirmed to have no adverse biological health effects on tissues.

In detail, the non-invasive high frequency patch microstrip technology focuses on the emission of electromagnetic waves into the tissues to provide accurate simulated changes of BP measurements. The new model first identifies the pulse transit time of a pulse wave, which is defined as the time difference of the variation of the distal and proximal transmission coefficient waveforms between two pairs of sensors with respect to varying artery coefficients. The distance between the two pair of antennas is equally set to half a wavelength. Second, PWV can be computed from pulse transit time (PTT) via PWV = L/PTT, where L is the distance between the two places of the propagation. The artery size variation was dependent on the artery before and after deformation to prove the presence of blood pressure due to the thickening and hardening of the artery. Finally, algorithms and regression models are discussed, so that PWV is related to SBP and DBP.

To verify the results of the proposed simulation model, the standard MK equation along with Hughes equation are performed while considering that the artery geometry is dynamic rather than static. The MK equation models a relationship between brachial artery size variation and the pulse wave velocity. The latter can be easily computed while assessing the arterial stiffness in the range of blood pressure. The use of the MK model shows excellent consistency with the proposed model so that PWV can be computed to assess the arterial strain in the range of blood pressure.

When the developed model and MK model do not hold any assumptions and take into consideration the thickness and radius of the artery change with any rise or drop in blood pressure levels, the pulse pressure propagation speed along with the artery stiffness also vary.

Thus, in this study, the PWV is directly related to arterial stiffness, an analytical model is established to yield a relation between blood pressure and pulse wave velocity. The results of both methods successfully demonstrate how the radius estimates, PTT, PWV and EM antenna transmission propagation characteristics can be used to estimate continuous BP non-invasively.

A significant advantage of the proposed design is that it requires no calibration for accurate results after each usage and is inexpensive, comfortable and highly accurate as it provides a desirable wearable option for home and office BP monitoring.

On the other hand, there are also some limitations with this new technique. First, it should be further validated through measurement on various arm sizes of healthy subjects and on subjects with different blood pressure levels and pathologies. In addition, the study must include different motion artifacts and tissue movements. Second, the relationship between PTT, the distance of the sensors and the patch-to-skin pressure should be investigated. Third, the proposed structure should be compared against the PPG structures to assess the advantages of each method. Fourth, towards realizing a complete device, the power consumption and the total size should be optimized. Finally, the BP validation protocol should be expanded, and various BP perturbations should be applied to a larger study cohort and in vivo experiments should be further studied, as the latter requires the presence and work of multidisciplinary medical fields including cardiologists and radiologists.

In summary, this simulated and real-time patient BP assessment provides the groundwork required to potentially develop a real-world non-invasive arterial pressure monitor system. The methodology of this system can be developed into a practical nano-wearable device that can provide timely and precise health-based care.

## Figures and Tables

**Table 1:** Dispersion characteristics of human tissue layers at 2.4 GHz.

Tissues	$\varepsilon_{r}$	*σ* (S/m)	*ρ* (Kg/m^3^)	*σ*_*mec*_ (W/K/m)
Skin	40.93	0.89	1100	0.293
Fat	5.34	0.08	1100	0.201
Muscle	55.19	1.49	1850	0.46
Bones	12.36	0.15	1020	0.41
Blood	59.19	2.11	1000	0.505

$\rho \;\left(\mathrm{Kg}/m^{3}\right):$ mass density; $\sigma_{mec}\;\left(W/K/m\right):$ mechanical compressibility; $\varepsilon_{r}:$ electrical permittivity; σ (S/m): conductivity.

**Table 2:** Optimized dimensions of the simulated patch antenna parameters.

Parameter	Value (mm)
Ls	35
Ws	35
Lp	26
Wp	20
Lg	35
Wg	4

Ls: Length of substrate; Ws: Width of substrate; Lp: Length of patch; Wp: Width of patch; Lg: Length of ground plane.

**Table 3:** Summary table showing the change in radiation characteristics of antenna with bending.

Cylindrical Bend [mm]	*S*_11_ [dB] at 2.4 GHz		Bandwidth (GHz)	
	Simulated	Measured	Simulated	Measured
R = 0	−47	−20	0.25	0.15
R = 45	−17	−13	0.2	0.25
R = 60	−17	−19	0.25	0.22
R = 75	−20	−20	0.25	0.5

**Table 4:** Simulated SAR in ten grams of arm tissue obtained from HFSS for the antennas placed at both sides of the human arm.

Power (mW)	SAR for 10 g (W/Kg)
1000	1.4917 × 10^3^
800	1.1933 × 10^3^
600	25.222
400	16.334
200	10.089
100	5.044
50	3.89

**Table 5:** Summary of estimated PTT, PWV and mean BP in the three studied cases.

Parameters	Case 1: h/R = 0.5	Case 2: h/R = 0.7	Case 3: h/R = 0.9
PTT (s)	0.8–1.8	0.15–0.75	0.1–1.6
PWV (m/s)	2.7–6.2	6.5–11	3–13
Mean BP	60–120	50–110	50–140
Systolic BP	80–120	100–140	100–140
Diastolic BP	20–60	40–80	90–130

PTT: Pulse Transit Time; PWV: Pulse Wave Velocity; h/R: thickness-to-radius.

**Table 6:** Summary of PWV and E estimates for different values of h/r ratios before artery deformation.

	Pulse Wave Velocity for Different Values of Different h/R Ratios							
E(kPa)	h/R = 0.08	h/R = 0.1	h/R = 0.12	h/R = 0.14	h/R = 0.16	h/R = 0.18	h/R = 0.2	h/R = 0.22
25	0.971	1.086	1.190	1.285	1.374	1.457	1.536	1.611
50	1.374	1.536	1.682	1.817	1.943	2.117	2.172	2.278
75	1.682	1.881	2.060	2.225	2.379	2.523	2.660	2.790
100	1.943	2.172	2.379	2.570	2.747	2.914	3.071	3.221
125	2.172	2.428	2.660	2.873	3.071	3.258	3.434	3.602
150	2.379	2.660	2.914	3.147	3.365	3.569	3.762	3.945
175	2.570	2.873	3.147	3.400	3.634	3.855	4.063	4.261
200	2.747	3.071	3.365	3.634	3.885	4.121	4.344	4.556
225	2.914	3.258	3.569	3.855	4.121	4.371	4.607	4.832
250	3.071	3.434	3.762	4.063	4.344	4.607	4.856	5.093

**Table 7:** Summary of PWV and E estimates for different values of thickness/radius ratios (h/r) after artery deformation due to blood pressure.

E (KPa)	h/R	PWV for h/R = 0.5	PWV for h/R = 0.6	PWV for h/R = 0.7	PWV for h/R = 0.8	PWV for h/R = 0.9
25	0.5	2.428	2.660	2.873	3.071	3.258
50	0.6	3.434	3.762	4.063	4.344	4.607
75	0.7	4.206	4.607	4.976	5.320	5.643
100	0.8	4.856	5.320	5.746	6.143	6.516
125	0.9	5.430	5.948	6.424	6.868	7.285
150		5.948	6.516	7.038	7.524	7.980
175		6.424	7.038	7.602	8.126	8.619
200		6.868	7.524	8.126	8.687	9.214
225		7.285	7.980	8.619	9.214	9.773
250		7.679	8.412	9.086	9.713	10.302

**Table 8:** Summary table comparing the two proposed methods for blood pressure estimation.

Parameters	Case 1: h/R = 0.5		Case 2: h/R = 0.7		Case 3: h/R = 0.9	
	Transmission Coefficient-PTT Method	Moens Korteweg Equation	Transmission Coefficient-PTT Method	Moens Korteweg Equations	Transmission Coefficient-PTT Method	Moens Korteweg Equation
PTT (s)	0.8–1.8	-	0.6–1.6	-	0.1–1.3	-
PWV (m/s)	2.7–6.2	2.5–7.5	6.5–11	3–9	3–13	3.5–10.5
Mean BP	60–120	30–100	50–110	30–130	70–140	40–160
SBP	80–120	80–130	100–140	80–150	100–140	90–165
DBP	20–60	55–80	50–90	55–85	50–90	55–95

## Data Availability

All data generated or analyzed during this study are included in this published article.
